# Editorial: dsRNA-based pesticides: production, development, and application technology

**DOI:** 10.3389/fbioe.2023.1197666

**Published:** 2023-04-13

**Authors:** Ruobing Guan, Tong Li, Guy Smagghe, Xuexia Miao, Haichao Li

**Affiliations:** ^1^ College of Plant Protection, Henan Agricultural University, Zhengzhou, China; ^2^ Key Laboratory of Insect Developmental and Evolutionary Biology, CAS Center for Excellence in Molecular Plant Sciences, Chinese Academy of Sciences, Shanghai, China; ^3^ Institute of Plant Protection, Henan Key Laboratory of Crop Pest Control, Key Laboratory of Integrated Pest Management on Crops in Southern Region of North China, Henan Academy of Agricultural Sciences, Zhengzhou, China; ^4^ Department Biology, Vrije Universiteit Brussels (VUB), Brussels, Belgium; ^5^ Department Plants and Crops, Ghent University, Ghent, Belgium; ^6^ Institute Entomology, Guizhou University, Guizhou, China

**Keywords:** dsRNA, RNA interference, biopesticides, crop protection, delivery

Since the discovery of RNA interference (RNAi) in 1998, a series of exciting results have been achieved in the field of applications (Fire et al., 1998). On 10 August 2018, the US Food and Drug Administration (FDA) approved ONPATTRO (patisiran), developed by Alnylam. It is used to treat neurological disorders (polyneuropathy) caused by hereditary transthyroxin protein amyloidosis (hATTR). It is also the first RNAi based drug to be approved worldwide, ushering in a new era in the development of targeted drugs using RNAi technology.

In the field of plant protection, RNAi has been demonstrated to hold considerable potential for pest control. On 15 June 2017, the United States Environmental Protection Agency (EPA) approved the world’s first insect-resistant transgenic corn MON87411 expressing double stranded RNA (dsRNA) targeting the DvSnf7 gene for control of rootworms, opening the third revolution in the history of pesticides (Christiaens et al., 2022; De Schutter et al., 2022). RNA biopesticides have the potential to control a variety of pests and diseases with environmental friendliness and high efficiency, which is a promising pest control strategy (Guan et al., 2021). Although some technical and application problems remain to be solved, cutting-edge research has proposed feasible solutions to many of these challenges. As technical and application problems are solved one by one the applications of dsRNA-based pesticide in the agricultural are expected to expand (Lucena-Leandro et al., 2022).

This Research Topic covers the most recent advances on the Research Topic of dsRNA synthesis, namely, the application method of RNAi pesticide and the scheme to promote the stability and efficiency of dsRNA. The successful cases of RNA pesticide, the obstacles to be overcome and feasible schemes are analyzed in order to realize the wide application of this technology in modern agriculture. Hough et al. reviewed dsRNA-based biocontrol having the potential to provide a species-selective and sustainable insect management strategy. At present, research on the large-scale manufacturing and mass delivery of dsRNA insecticides is in its infancy, and all potential methods present challenges. Specific target insects or application environments require different and more appropriate approaches to address, and have additional challenges related to product characterization, quantification, quality control, and regulatory approval that need to be addressed. Furthermore, it is unlikely that a universal method exists that is effective for the control of all conceivable target species, and production and delivery methods will have to be tailored accordingly (Hough et al.). He et al. reviewed the methods for mass production of dsRNA, the approaches of exogenous application of dsRNA in the field, and the fate of dsRNA after application. It will be obvious that the development of dsRNA pesticides to improve the control efficiency of target pests is of great importance in the commercialization of dsRNA pesticides. Also, adequate risk assessment is required to minimize off-target risks for non-target organisms and develop handling recommendations in the field (He et al.). Nie et al. summarizes the key harmful genes associated with aquatic pathogens (viruses, bacteria, and parasites) and provides potential targets for the preparation of dsRNA (Nie et al.).

Delivery of dsRNA-based pesticides is another important Research Topic addressed in this Research Topic. Yang et al. reviews the recent research progress on nanomaterials that can be used to improve the environmental stability of dsRNA, and discusses the advantages and limitations of different nanomaterials when combined with dsRNA. Overall, nanotechnology combined with dsRNA has many advantages and can provide new avenues for pest control. However, many key Research Topic, such as biosafety and environmental release of dsRNA still need to be considered during the research and development process (Yang et al.). Furthermore, Li et al. reviewed the development of dsRNA-expressing transgenic plants, the status and advantages of deploying these products for pest management, as well as the future research directions and existing problems in production and commercialization of these products (Li et al.).

In addition, this Research Topic also accepted 3 original research papers. Research and analysis were carried out on the genes of *Bemisia tabaci* BtGR11, *Nilaparvata lugens* delta (dl) and jagged (jag), and *Helicoverpa armigera* glyceraldehyde-3-phosphate dehydrogenase (GAPDH). The conclusions showed that these genes can be used as targets for pest control based on RNAi technology (Li et al.; Yang et al.; Zhao et al.).

The use of chemical pesticides has greatly increased agricultural productivity, but excessive use can damage agroecosystems and jeopardize human health and food security (Shaffer, 2020). DsRNA-based pesticides exploit RNAi-mediated gene suppression to disrupt growth and development of target pests, ultimately killing them (Zhu and Palli, 2020). This new generation of pesticides will likely revolutionize the field of pest management by enhancing control while reducing the need for chemical pesticides. Specifically, dsRNA-based pesticides that use water as carrier can significantly reduce the use of organic chemicals, such as solvents and additives, that are used in chemical pesticides. Moreover, dsRNA-based pesticides can also play an important role in delaying the evolution of resistance to chemical pesticides (Zotti et al., 2018; Mat Jalaluddin et al., 2019; Kunte et al., 2020; Lucena-Leandro et al., 2022). Based on many studies, dsRNA-based pesticides with their unique mode of action, gene silencing, will likely solve many problems related to environmental protection and food safety that traditional pesticides cannot. In particular, dsRNA-based pesticides offer promising solutions for controlling other important pests, such as nematodes, aphids, and whiteflies, *etc.*, undoubtedly provide a practical control strategy for a more sustainable food production with safety to human and environment to feed a growing world population.
